# *Escherichia coli* Alpha-Hemolysin HlyA Induces Host Cell Polarity Changes, Epithelial Barrier Dysfunction and Cell Detachment in Human Colon Carcinoma Caco-2 Cell Model via PTEN-Dependent Dysregulation of Cell Junctions

**DOI:** 10.3390/toxins13080520

**Published:** 2021-07-26

**Authors:** Emanuel Schulz, Michael Schumann, Martina Schneemann, Violaine Dony, Anja Fromm, Oliver Nagel, Jörg-Dieter Schulzke, Roland Bücker

**Affiliations:** 1 Department of Gastroenterology, Infectious Diseases and Rheumatology, Charité—Universitätsmedizin Berlin, Hindenburgdamm 30, 12203 Berlin, Germany; emanuel.schulz@charite.de (E.S.); michael.schumann@charite.de (M.S.); violaine.dony@charite.de (V.D.); 2 Junior Clinician Scientist Program, Biomedical Innovation Academy, Berlin Institute of Health at Charité—Universitätsmedizin Berlin, Charitéplatz 1, 10117 Berlin, Germany; 3 Clinical Physiology/Nutritional Medicine, Charité—Universitätsmedizin Berlin, Hindenburgdamm 30, 12203 Berlin, Germany; martina.schneemann@charite.de (M.S.); anja.fromm@charite.de (A.F.); oliver.nagel@fu-berlin.de (O.N.); joerg.schulzke@charite.de (J.-D.S.)

**Keywords:** hemolysin, *Escherichia coli*, epithelial barrier, cell polarity, cell detachment, leaky gut, colon carcinoma, PTEN, phosphatidylinositol, tight junction

## Abstract

*Escherichia coli* (*E. coli*) of the B2 phylotype reside in human and animal intestines. The bacteria possess pathogenicity factors such as α-hemolysin (HlyA) that can induce intestinal epithelial leaks. We addressed the questions which host cell processes were dysregulated by *E. coli* HlyA that can potentiate intestinal diseases. The colon carcinoma cell line Caco-2 was infected by HlyA^+^ *E. coli*. Cell polarity regulation was analyzed by live cell imaging for the phosphatidylinositol-4,5-bisphosphate (PIP2) abundance. In Caco-2 monolayers, transepithelial electrical resistance was measured for characterization of barrier function. Cell proliferation and separation were assessed microscopically. Epithelial regulation and cell signaling were analyzed by RNA-Seq and Ingenuity Pathway Analysis (IPA). Our main findings from *E. coli* HlyA toxinogenicity in the colon carcinoma cell line are that (i) PIP2 at the membrane decrease, (ii) PTEN (phosphatase and tensin homolog) inhibition leads to cell polarity changes, (iii) epithelial leakiness follows these polarity changes by disruption of cell junctions and (iv) epithelial cell detachment increases. HlyA affected pathways, e.g., the PTEN and metastasis signaling, were identified by RNA-Seq bioinformatics calculations in IPA. In conclusion, HlyA affects cell polarity, thereby inducing epithelial barrier dysfunction due to defective tight junctions and *focal leak* induction as an exemplary mechanism for *leaky gut*.

## 1. Introduction

The molecular crosstalk between the intestinal epithelium and luminal bacteria is a pivotal event in bacterial infections of the gastrointestinal tract. Many *Enterobacteriaceae* show adhering and invading properties that reflect their virulent phenotype in intestinal infections. Changes of the composition of the gut microbiota with high abundance of *Enterobacteriaceae* can be seen both in chronic intestinal inflammation, e.g., intestinal bowel disease (IBD) and in colitis models [1,2]. Thus, this bacterial family and especially *Escherichia coli* (*E. coli*) is gaining high interest for current research approaches in human and veterinary medicine. Even species from the *Enterobacteriaceae* family that used to be considered as commensal bacteria in the gut, e.g., the uropathogenic *E. coli* (UPEC), were shown to have a potential pathogenic impact on the intestinal epithelium. *Focal leaks* in the colonic epithelial barrier were described in vitro on HT-29/B6 cells induced by the β-barrel pore-forming toxin (PFT) α-hemolysin (HlyA) of UPEC [3]. Further studies could depict increased transepithelial permeability for macromolecules and intestinal inflammation due to the HlyA^+^ *E. coli* 536 infection in an in vivo mouse model [4]. Moreover, HlyA-producing bacteria were shown to be present in a higher abundance in active ulcerative colitis within the human colon mucosa [4]. These results are in line with different studies, which associate intestinal colonization by *E. coli* of the phylogenetic group B2 and D with the pathogenesis of ulcerative colitis [5]. Whereas low numbers of HlyA-producing bacteria can also be found in healthy controls, data on the abundance of UPEC in intestinal diseases other than IBD are sparse. The regulation of epithelial barrier function during the infection with HlyA^+^ *E.coli* by changes in tight junction (TJ) protein distribution was shown recently in porcine colon specimens [6]. How the TJ proteins occludin or members of the claudin family are regulated by HlyA via intracellular signaling is still unknown. Moreover, the initial induction and further pathogenesis of epithelial *focal leaks* by HlyA is not well-described. However, it was shown that *E. coli* HlyA directly initiate intestinal barrier dysfunction, contributing on the one hand to diarrhea driven by a *leak flux* mechanism and on the other hand to the *leaky gut* phenomenon, allowing the entry of luminal antigens into the submucosa, which induce inflammation and subsequently cytokine-accelerated epithelial barrier disruption [4]. These effects were assigned to calcium (Ca^2+^) signaling in the epithelial cells [7], since the HlyA pores in the host cell membrane mediate Ca^2+^ influx through the pores [8,9].

Analysis on cell signaling in infected bladder cells with HlyA-harboring UPEC or HlyA alone lead to dephosphorylation of AKT/Protein kinase B (PKB), a key regulator of inflammatory responses, host cell survival, proliferation and metabolism [9]. HlyA, but not other pore-forming toxins, induces a degradation of scaffolding and signaling proteins like paxillin and PAK-1 [10]. Infection with HlyA-harboring UPEC leads to exfoliation of bladder epithelial cells. Whether this is due to the pathomechanism of the bacterium or part of the host defense mechanism to purge the infected cells so far remains unclear.

Since HlyA was shown to contribute to epithelial damage in intestinal diseases as IBD (i.e., ulcerative colitis) and other inflammatory diseases (i.e., cystitis), the higher toxinogenic potential of the HlyA^+^ *E. coli* could also depend on the invasion of bacteria in the epithelial layer. Therefore, it is of importance to identify host cell signals that are dysregulated by either toxins or the bacterial contact, mediating the epithelial damage and subsequently disease. In particular, epithelial cell polarity regulation, i.e., phosphoinositide (phosphatidylinositol phosphate (PIP)) signaling, triggered by the bacteria could in fact be a first step in the pathogenesis of the infection, inducing damage of the intestinal mucosa.

The Phosphatase and Tensin homolog deleted on chromosome ten (PTEN) is a phosphatase playing a role in regulation of cell polarity and tumor suppression. PTEN was described to localize to the plasma membrane, the cytosol as well as the nucleus. It dephosphorylates phosphatidylinositol-3,4,5-trisphosphate (PIP3), thereby suppressing AKT/PKB activation. In the process of epithelial morphogenesis and polarization, PTEN localizes in the apical membrane, enriching this compartment in phosphatidylinositol-4,5-bisphosphate (PIP2). PIP2 sums up for about 1% of the lipids in the plasma membrane. Nevertheless, as a source of three important second messengers, it is a key substrate in multiple membrane functions, e.g., endocytosis, exocytosis, enzyme activation and actin cytoskeleton organization, to name but a few [11].

Besides the PIP3-dephosphorylation, PTEN has a plethora of functions. For instance, it also regulates Ca^2+^-release from the endoplasmatic reticulum and mitochondria-associated membranes and modulates the cell cycle. Loss of PTEN function increases the amount of PIP3 in the plasma membrane and is associated with cell polarity changes, cell growth, cell migration and prolonged cell survival. In experimental studies, with inhibition of the PTEN pathway or deregulation of PTEN binding partners in intestinal epithelial cells, it was proposed that PTEN plays a role in colorectal cancer progression [12]. In many types of cancer, PTEN expression was found to be altered [13]. In colorectal cancers, PTEN alterations are associated with microsatellite instability, BRAF mutations and advanced metastases [14]. Loss of PTEN gene function leads to disordered polarization of intestinal cells [12]. Furthermore, PTEN loss in Caco-2 cells leads to higher Cdc42 levels and increased cell motility and invasion properties with upregulated expression of matrix metalloproteinases (MMP2, MMP9) and urokinase plasminogen activator (uPA) [12]. Interestingly, disruption of PTEN in the intestinal epithelium of IL-10^−/−^ mice speeds up the onset and increases the severity of colitis [15]. Reduced PTEN activity is known to increase cell migration in cancer cell lines [12]. Interestingly, cell protrusions from the epithelial layer, leading to cell shedding, have also been described after HlyA infection in both bladder and intestinal epithelium [7,16,17]. Single cell separation or collective cell migration like in metastasis could be associated to polarity changes and PTEN regulation and could be linked to pore-forming toxins like HlyA, as one aspect of intestinal pathology.

In order to identify pathomechanisms that might also serve as targets for future treatment options, we investigated the influence of HlyA from *E. coli* 536 on cell polarity and epithelial barrier function of human colon carcinoma Caco-2 cells, with respect to the molecular targets and its subcellular distribution during infection. Since it was shown in other bacterial infection models that cell polarity and PIP signaling were affected during the first steps of infection [18], we hypothesized that HlyA^+^ *E. coli* influences cell polarity in a similar way. In order to assess the abundance of PIP2, we used a green fluorescent protein (GFP)-bound biosensor that targets PIP2 by a Pleckstrin Homology (PH) domain. The PH domain is part of the membrane targeting and activation of phospholipase Cδ (PLCδ) domain connected to a homology binding domain of AKT (GFP-PLCδ-PH construct). Moreover, we aimed at characterizing the concurrent effect on epithelial barrier function in the first hours of infection of Caco-2 monolayers and the cellular impairment during the course of the HlyA^+^ *E. coli* infection.

## 2. Results

### 2.1. Cell Polarity. Depletion of Host Cell Membrane Phosphatidylinositol 4,5-Bisphosphate (PIP2) in Single, Non-Polarized Caco-2 Cells after HlyA^+^ Escherichia coli Infection Point to Changes in Polarity of Involved Epithelia

To analyze host cell membrane changes in PIP signaling as early events of the infection, we microscopically assessed single bacteria in proximity to single Caco-2 cells. For visualization of HlyA^+^ *E. coli*-induced polarization changes, Caco-2 cells have been transfected with GFP-PLC-PH prior to infection. The selective binding of the PH domain to PIP2 is used as a fluorescent biosensor (green fluorescent protein, GFP) to monitor changes or local differences in the concentration of plasma membrane PIP2. To resolve spatio-temporal dynamics of PIP2 in GFP-PH-expressing epithelial cells, we performed confocal laser-scanning microscopy (LSM)-based live cell imaging.

**Figure 1:**
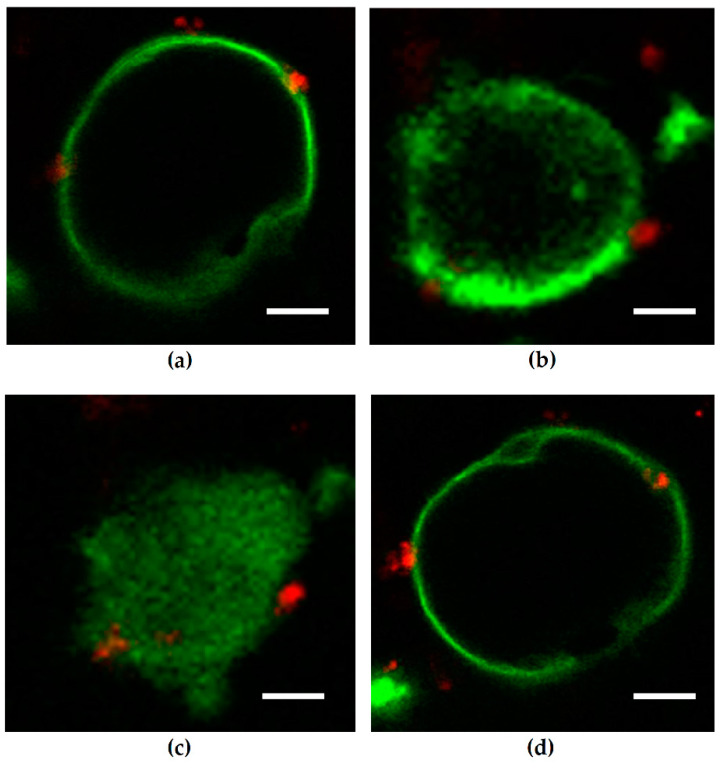
Phosphatidylinositol 4,5-bisphosphate (PIP2) reporter signals in non-polarized Caco-2 cells (GPF-PLC-PH-transfected) after *Escherichia coli* 536 infection. Confocal laser-scanning microscopy (LSM) from live cell imaging experiments. Green microscopic signal from reporter GFP-PH-domain bound to PIP2 (PH-domain as reporter for PIP2). Bacteria are displayed red by Hoechst staining. (**a**) Control with sharp PIP2 reporter signals (green) at the membrane after 30 min. Cells were infected with the isogenic hemolysin-deficient mutant (HDM) of *E. coli* 536 as attached control organism (red). (**b**) Infection with the hemolysin-positive *E. coli* 536 (red) led to a loss of the microscopic PIP2 reporter signal (green) from the plasma membrane to an intracellular compartment within 30 min. (**c**) After one hour of infection with *E. coli* 536 the delocalization of PIP2 signal off the membrane towards intracellular was pronounced. (**d**) In control experiments with infection of Caco-2 cells by HDM, the PIP2 signal remains at the host cell membrane even after 3 h p.i. Cells were observed by LSM in Lab-Tek II chamber slides and were infected with a multiplicity of infection (MOI) of 50. Bar = 5 µm.

In untreated controls, the microscopic GFP-signal displaying PIP2 was evenly distributed in the cell membrane, which was not altered after control infection with the isogenic hemolysin-deficient mutant (HDM) of *E. coli* 536 (Figure 1a). On the contrary, infection with HlyA-producing *E. coli* 536 induced a delocalization of the microscopic GFP-signal from the membrane (Figure 1b). The redistribution of the GFP-PLC-PH domain from the cell membrane into the cytosol occurred within 30 min after exposure of the epithelial cells with bacteria and was pronounced 60 min post infection (p.i.) (Figure 1c). Even hours p.i., cells infected with the HDM did not show changes in the localization of the GFP-PLC-PH-signal. Similarly to untreated control cells, the cell membrane revealed a sharp localization of the microscopic PIP2 reporter signal (Figure 1d). The vanishing of the GFP-signal in the *E. coli* 536-infected cells can be interpreted as a loss of PIP2 in the plasma membrane or, alternatively, represents the phosphorylation from PIP2 to PIP3, to which the PH-domain does not bind. Thus, the abundance of PIP2 in the membrane declines right after infection.

**Figure 2:**
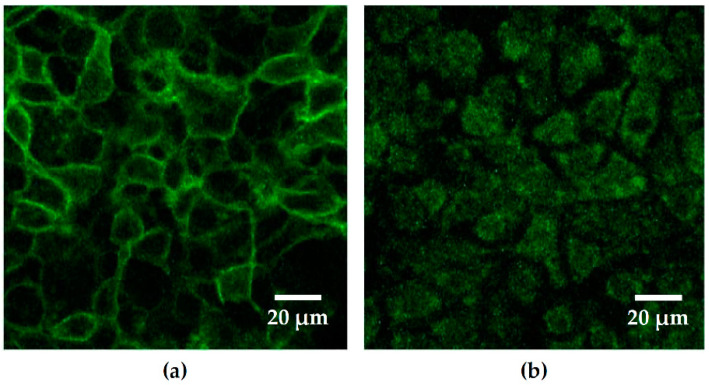
Confluent Caco-2 monolayers in response to hemolysin (HlyA). PIP2 reporter signal of a polarized Caco-2 cell monolayer transfected with GFP-PLC-PH-domain. The green fluorescent signal discloses the localization of PIP2. (**a**) As control condition, the Caco-2 monolayers were infected with the hemolysin-deficient mutant (HDM) of *E. coli* 536. Here, the green GFP-PH signal represents the PIP2 abundance at or in close proximity to the cell membranes. (**b**) Caco-2 cells infected with HlyA-producing *E. coli* 536 showed a redistribution of PIP2 reporter signals off the cell membrane to an intracellular compartment. MOI 100, 2 h post infection.

Moreover, these effects were also observed in confluent Caco-2 cell monolayers, stably transfected with the GFP-PLC-PH vector. These polarized monolayers develop a stable apical-basolateral barrier. After infection with HlyA-secreting *E. coli* 536, again a delocalization of the GFP signal could be observed, but not in control conditions and not after infection with the HDM (Figure 2). Hence, also in the confluent polarized cell monolayers, the PIP2 abundance in the apical membrane decreased, and the microscopic GFP signal switched to intracellular localization after infection with *E. coli* 536, revealing that the PIP2 content in the apical plasma membrane was decreased.

### 2.2. Epithelial Barrier. Decrease in Transepithelial Electrical Resistance (TER) of Polarized Caco2-Cell Monolayers (GPF-PLC-PH-Transfected) after HlyA^+^ Escherichia coli Infection Concurrent to the Change in PIP2 Abundance

The effect of HlyA on epithelial barrier function has previously been shown by our group with transepithelial electrical resistance (TER) measurements in the human colon cell model HT-29/B6 in vitro and in the infected mouse or porcine colon ex vivo [3,4,6,7]. To combine the functional models with our Caco-2 cell model on subcellular PIP2 distribution, we selected a Caco-2 clone, which was stably transfected with the GPF-PLC-PH vector, showing appropriate GFP signals, and which formed confluent monolayers on PCF filter supports for TER measurements and could serve as infection model. These Caco-2-GFP-PLC-PH monolayers displayed an even PIP2 distribution under confocal LSM examination (Figure 2a). TER measurements of the confluent monolayers revealed stable epithelial resistances comparable to wild type Caco-2 control conditions, with TER values ranging between 500 and 900 Ohm∙cm^2^.

**Figure 3:**
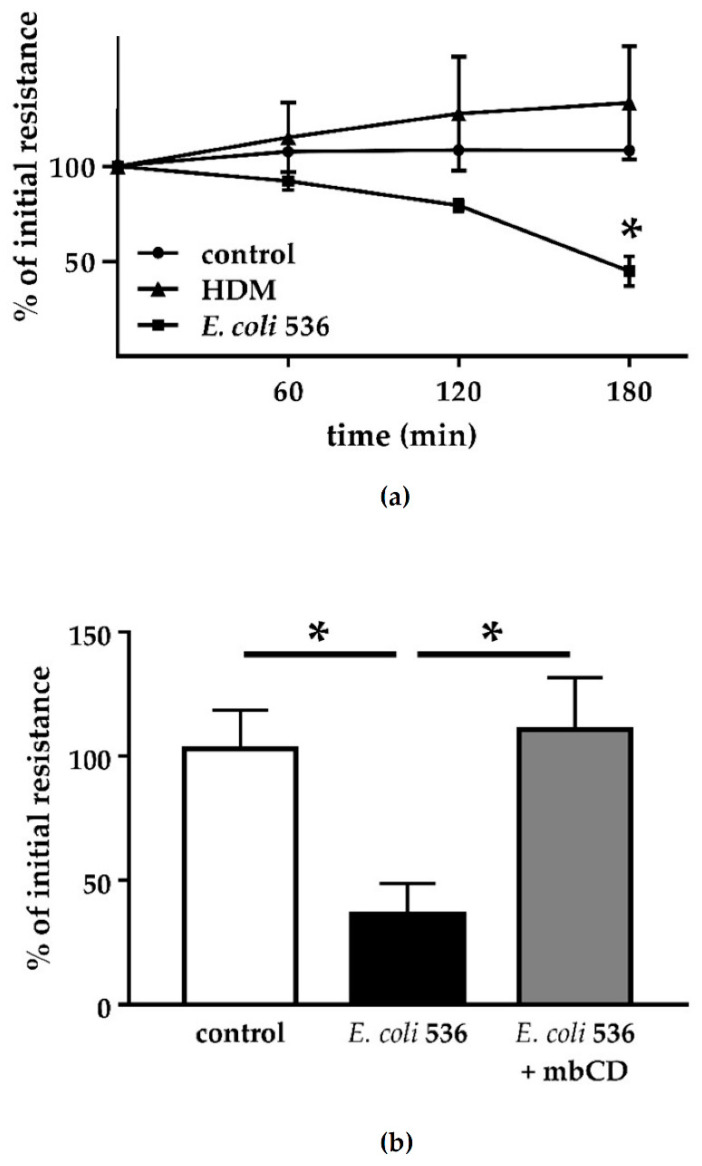
Epithelial barrier function in Caco-2 cell monolayers after challenge with HlyA-producing *E. coli*. Transepithelial electrical resistance was measured in transfected Caco-2 monolayers, representing epithelial barrier function. (**a**) Infection with *E. coli* 536 (squares) affects TER within 3 h, whereas the hemolysin-deficient mutant (HDM) failed to affect TER (triangles); * *p* < 0.05, 536 vs. HDM, n = 3. (**b**) Depletion of membrane cholesterol by methyl-β-cyclodextrin (mbCD, 5 mM) prevented the HlyA^+^ *E. coli* 536-dependent TER drop. TER values are corrected to baseline values of control Caco-2 cells (100%). * *p* < 0.05, n = 3.

In this Caco-2 model, TER was reduced to 50% after infection with *E. coli* 536 when compared to the control using infection with HDM (Figure 3). Using infection doses between MOI 50 and 100, the drop in TER became significant in the first 2 to 3 h p.i. with *E. coli* 536 (Figure 3a), in the time frame of the PIP2 changes. A difference between HDM-infected cells and non-infected cells was not detected (Figure 3a). As previously reported in HT-29/B6 cells, the TER drop was reproducible after exposing the epithelial cells with HlyA-containing supernatant, whereas supernatant of HDM did not affect TER [3]. The same was true for the non-transfected Caco-2 cells (data not shown).

As pore-forming toxins like HlyA are thought to insert receptor-independently into cholesterol-rich regions of the plasma membrane (lipid rafts) [19], we pretreated the apical membrane of our Caco-2 cell monolayers with methyl-β-cyclodextrin (mbCD) in order to deplete cholesterols from the host cell membrane. In mbCD-pretreated cells, the decrease in TER after infection with HlyA^+^ *E. coli* 536 was blocked (Figure 3b). Thus, results of these experiments making use of HDM, bacterial supernatants, or mbCD allow the conclusion that the TER and PIP2 effects rather depend on the toxin HlyA and not on the bacterial background. Moreover, we uncovered that the HlyA effect can only be alleviated when its integration into the host cell membrane is disrupted, comparable to previous results from our group in experiments after addition of zinc [6,7]. These are the only inhibition experiments that worked in the initial phase of HlyA insertion. Contrary to that, various inhibitors of cell signaling failed to counter-regulate HlyA barrier defects (see Section 2.4). These experiments provide evidence that the barrier dysfunction by *E. coli* 536 depends on the presence and membrane insertion of HlyA.

### 2.3. Cell Junctions and Polarity. After Infection with HlyA^+^ E. coli, Occludin Is Dislocated from Tight Junctions and PTEN Is Redistributed from the Cell Membrane Together with a Rearrangement of E-Cadherin

The correlation of structural and barrier defects caused by HlyA was first assigned by our group along with the induction of epithelial *focal leaks* in colonic monolayers in vitro [3] and in mouse colon in vivo [4]. However, the barrier dysfunction may also involve the TJs and the adherens junctions (AJs) as initial targets for the process that later leads to cell detachment.

**Figure 4:**
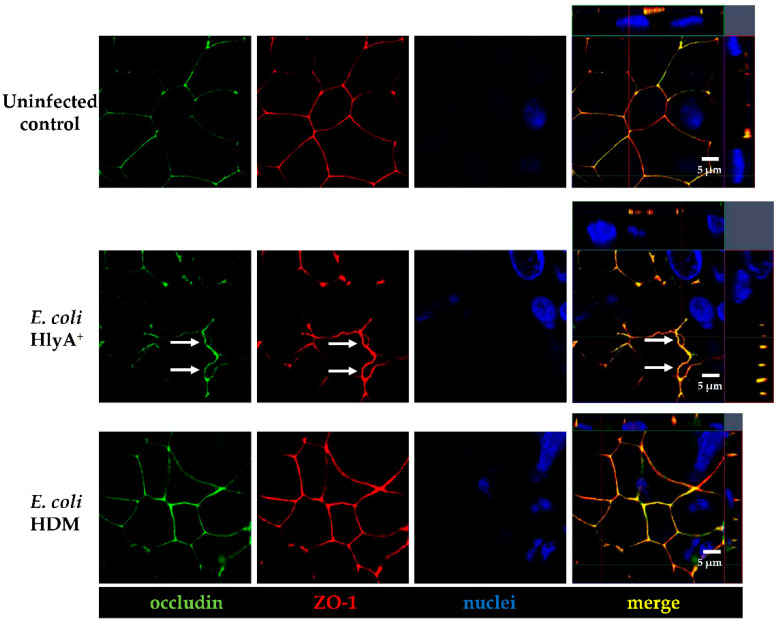
Subcellular tight junction distribution in HlyA^+^ *E. coli*-infected Caco-2 monolayers in apical view by confocal microscopy 3 h p.i. (**Upper row**) Uninfected control cells; the TJ meshwork pattern appears with sharp cell–cell contacts and colocalization of occludin (green) together with ZO-1 (red) is displayed as merge signal (yellow) in orthographical display of Z-stacks on the right side. Nuclei are stained using DAPI (blue). (**Middle row**) Infection with HlyA-secreting *E. coli* induces division of cell membranes of two neighboring cells, indicated by signals of occludin and ZO-1 being retracted together from bicellular connections (white arrows). (**Lower row**) Control infection with HlyA^−^ *E. coli* HDM revealed a proper tight junction meshwork comparable to uninfected controls.

To prove if the early barrier defect by HlyA, detected here by the TER decrease, was caused by alterations of the barrier-forming TJ proteins, we performed immunostainings of occludin and zonula occludens protein-1 (ZO-1) in *E. coli* 536-infected Caco-2 cells (Figure 4). LSM imaging revealed no general disturbance in TJ protein localization. Nevertheless, a detachment of cell-cell contacts at the TJs domain was observed after infection with HlyA-secreting *E. coli*, whereas in HDM controls or in uninfected control Caco-2 cells a sharp TJ meshwork pattern with a proper colocalization of occludin with ZO-1 was visible (Figure 4). This opening of bicellular TJs might be an explanation for the early TER decrease and furthermore for the initial impact on the induction of *focal leaks*.

**Figure 5:**
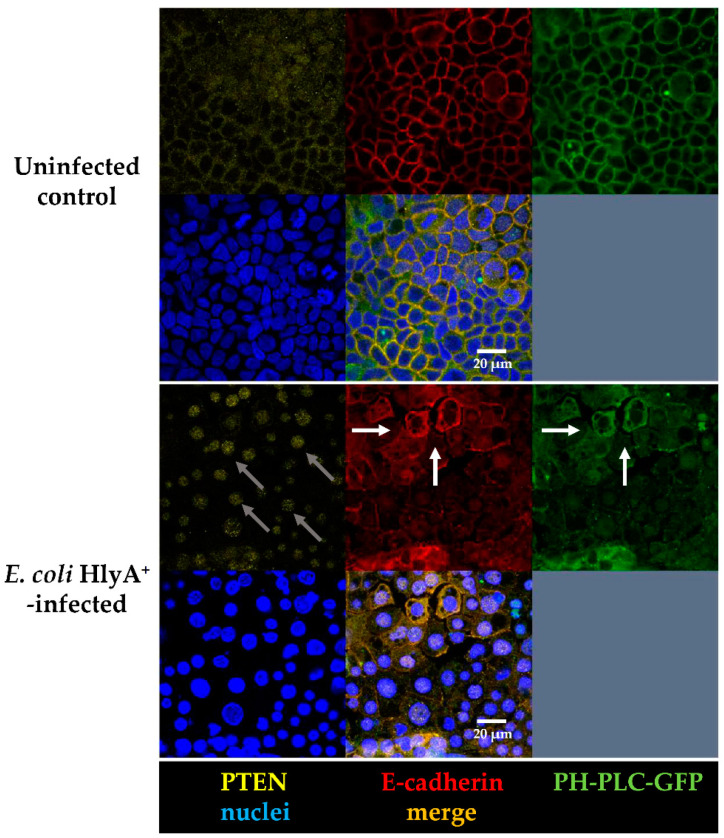
Confocal micrographs of Caco-2 monolayers. Rearrangement of PTEN and E-cadherin together with cell polarity changes in PIP2 abundance after HlyA^+^ *E. coli* 536 infection 3 h p.i. (**First panel**) Control Caco-2 monolayers apical view on the distribution of PTEN (yellow), E-cadherin (red), GFP-PH domain (green), nuclei (blue), and merge of all channels in the lower middle frame. (**Second panel**) *E. coli* 536-infected monolayers with simultaneous distribution changes of PTEN, E-cadherin and GFP-PH signals. White arrows indicate detachment of epithelial cells in proximity to the apical membrane in the adherens junction. Grey arrows indicate PTEN localization in the nuclei.

Immunostainings of the AJ protein E-cadherin revealed an altered distribution in Caco-2 cells (Figure 5). In non-infected controls and control cells infected with HDM, E-cadherin was found in a submembranous compartment close to the lateral cell membranes. After infection with HlyA^+^ *E. coli* 536, E-cadherin was detected partly at the AJ and additionally in the cytosol (Figure 5). In uninfected control monolayers, PTEN was localized to the plasma membrane at cell–cell contacts and to a lower extent also to the cytosol and the nucleus (Figure 5). However, after infection with *E. coli* 536, PTEN signals vanished from the plasma membrane accumulating in the nuclei (Figure 5). As a reason for the polarity changes in PIP signaling, the inhibition of the PTEN signaling pathway is plausible, which is further analyzed in Section 2.7 on differential gene expression profiles.

### 2.4. Polarity and Barrier Function. PTEN Inhibition Leads to Perturbation of the Epithelial Barrier

Since PIP- and PTEN- signaling is affected by HlyA, we aimed to test the impact of PTEN signaling on barrier function and wanted to identify further possibly involved pathways that could lead to polarity changes or destabilization of TJs or AJs and finally to cell detachment. Inhibitory experiments were carried out during infection with HlyA^+^ *E. coli* 536 in the Caco-2 monolayers, and the TER was recorded continuously.

**Figure 6:**
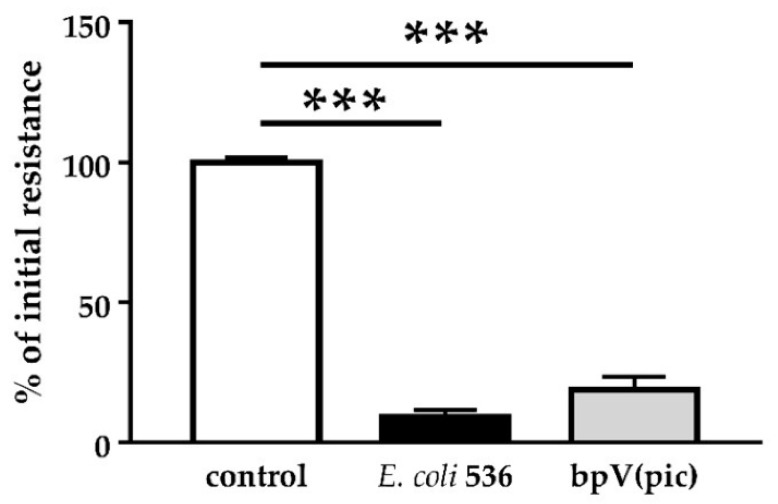
PTEN inhibition causes a decrease in epithelial resistance. Application of 1 µM of the PTEN inhibitor bpV(pic) to Caco-2 monolayers revealed a drop in resistance within 2 h and likewise *E. coli* 536 infection with a similar time frame. *** *p* < 0.001, n = 3.

Both GFP-PLC-PH signal and PTEN localization changes in Section 2.3 suggested an involvement of PTEN in the process leading to barrier disruption. Subsequently, we examined the effect of PTEN inhibition in our cell culture model with bpV(pic). In the GFP-PLC-PH-transfected Caco-2 cells, we detected a strong and rapid decrease of TER immediately after incubation with the PTEN inhibitor bpV(pic) (Figure 6). In parallel, experiments with various kinase inhibitors did not result in a recovery of the HlyA-induced barrier damage. This included inhibition of PI3K (Ly294002), PLC (U73122), MLCK (ML-7, H7), PKC (H7, Calphostin C) or ERK 1/2 (U0126) in Caco-2 monolayers as well as HT-29/B6 cells (data not shown).

### 2.5. Relation between Polarity and Cell Movement. Both the PTEN Inhibitor bpV(pic) and E. coli 536 Promote Cell Separation

**Figure 7:**
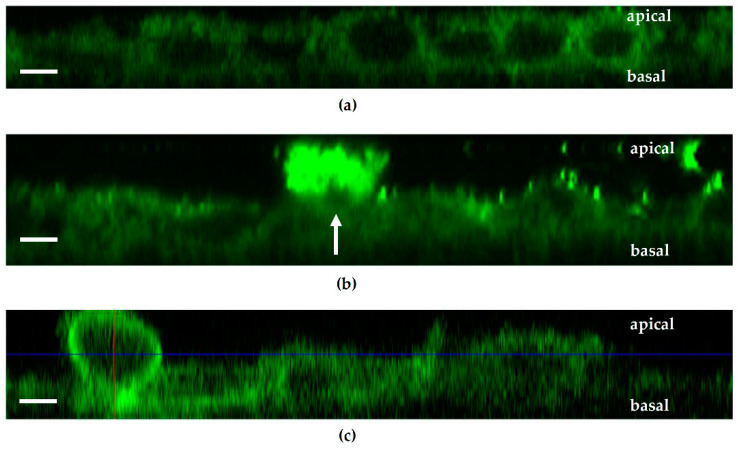
Cell detachment after PTEN inhibition or HlyA treatment within the first 2 h. Lateral view on Caco-2 monolayers in live cell imaging. (**a**) Untreated control. (**b**) *E. coli* infection. White arrow indicates cell exfoliation after HlyA^+^ *E. coli* infection. (**c**) PTEN inhibitor bpV(pic)-treated monolayers. The crossed lines in bpV(pic) treated cells indicate a cell protrusion right before shedding into the apical compartment. Bar = 5 µm.

In our experiments, we observed cells leaving the epithelial lining and moving within the epithelial level to a more apical position both after HlyA^+^ *E. coli* infection and after bpV(pic) treatment. The lateral view on the Caco-2 cell monolayers by microscopic live cell imaging revealed single cells detaching from the cell bond in HlyA^+^ *E. coli*-infected or in PTEN inhibitor bpV(pic)-treated cell monolayers (Figure 7). The cells move to the apical compartment. Thus, a shedding of cells seemed to be enhanced by *E. coli* HlyA (see Section 2.6). The mechanism for such a cell shedding seems to be dependent on PTEN inhibition. The *focal leaks* observed at later time points revealed of course a loss of several cells, whereas in this early stage of infection, we rather observed single cell loss as initial event of the HlyA effect.

### 2.6. Cell Proliferation and Separation. Escherichia coli HlyA Induces Cell Proliferation and Increases the Number of Separated Viable Colon Carcinoma Caco-2 Cells

**Figure 8:**
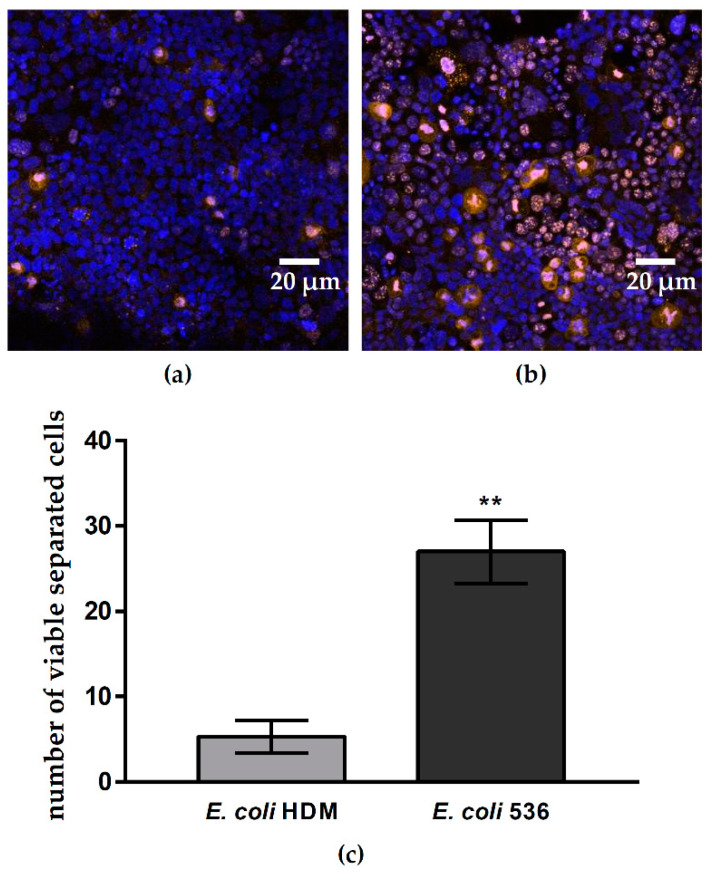
Caco-2 cell proliferation and cell separation after *E. coli* HlyA. Monolayers treated with *E. coli* 536 or control conditions were analyzed for cell proliferation and counted for viable shedded cells. (**a**) Microscopic low-power field of a counting area with Caco-2 cell nuclei stained blue and proliferating cells immunostained in orange using Ki67 antibody (nuclei, blue). (**b**) Ki-67-positive cells (orange) in Caco-2 monolayers and nuclei (blue) after HlyA^+^ *E. coli* 536 infection for quantification of the proliferation rate. (**c**) Number of viable cells shedded from the infected Caco-2 monolayers. Disseminated Caco-2 cells from the supernatant of infection experiments were seeded on a fresh 24-well. Numbers of viable cells were determined microscopically. Dead cells were excluded by trypan blue staining. The number of dead cells in all groups ranged from 1 to 8 per well without reaching statistical difference. ** *p* < 0.01, n = 6–12, Student’s t-test for viable separated Caco-2 cells after *E. coli* 536 vs. HDM.

As both PTEN and PIP2 are known to play a central role in cell proliferation, we used Ki-67 staining as marker for cell proliferation during *E. coli* HlyA^+^-infection. The overall proportion of Ki-67-positive cells was ten times higher after infection with HlyA^+^ *E. coli* 536 compared to control monolayers (56% ± 7% Ki-67-positive cells in infected monolayers vs. 5% ± 1% in control monolayers, *p* < 0.001, n = 9) (Figure 8a,b). We then analyzed if and how many epithelial cells come off from the monolayers after infection, as already qualitatively described before [7], and if these cells were still viable. Caco-2 cells serve as model for the intestinal epithelium and are originally a colon carcinoma cell line. Thus, the separation of these colon carcinoma cells may also have relevance for development of metastasis (dissemination). Cell that are shedded from monolayers into the supernatant were seeded in 24-wells and counted. The number of cells that had left the cell monolayer into the supernatant increased 5-fold after infection with HlyA^+^ *E. coli* 536 when compared to HDM control infection (Figure 8c). The counted cells attached to the surface of a new 24-well were still viable 24 h after infection in the trypan blue test.

### 2.7. Differential RNA Expression and Canonical Pathways. Pathway Analysis by Bioinformatics from RNA-Sequencing Datasets of Caco-2 Monolayers Infected by HlyA^+^ Escherichia coli

After 5 h of infection, the Caco-2 monolayers were prepared for total RNA extraction and subsequently processed for RNA library preparation and sequencing. Data are deposited in NCBI’s Gene Expression Omnibus (GEO ID GSE169213). Differential gene expression analysis revealed mRNA expression profiles with 20,674 genes that were differentially expressed after *E. coli* 536 infection compared to uninfected controls; 18,242 were upregulated and 2432 downregulated. The comparison of *E. coli* 536 with HDM revealed 19,890 genes to be differentially expressed, pointing to a major contribution of HlyA in the expression changes.

**Figure 9:**
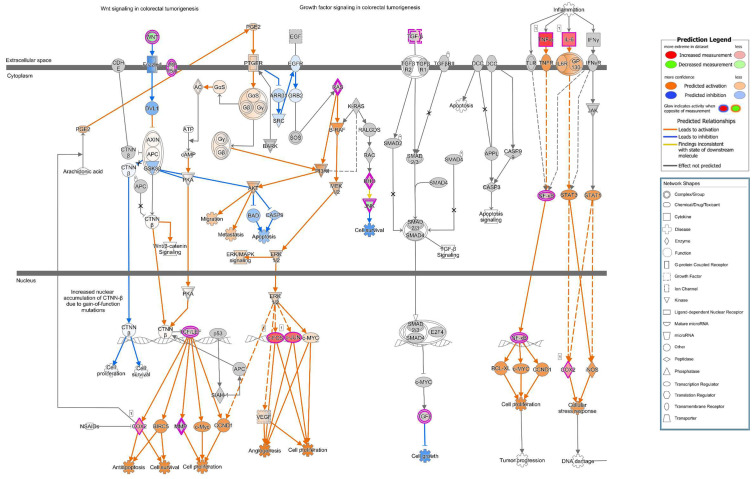
Canonical pathways activated in *E. coli* 536-infected Caco-2 monolayers. RNA-Seq of Caco-2 cells and subsequent bioinformatic pathway analysis by the Ingenuity Pathway Analysis software (IPA) revealed “Colorectal cancer metastasis signaling” to be activated after infection. The symbol legend is embedded into the figure. The dataset from the experimental infection is overlayed in a pre-existing pattern of pathways. Orange color indicates activation and blue color indicates inhibition patterns. Grey nodes indicate no sufficient data or inconsistent data for a clear prediction. The pathway was visualized using the molecule prediction tool of IPA. Dotted lines indicate that the connection is not direct, and some nodes (not depicted) might be affected in between. In this pathway analysis, following the arrows for inhibition in the Wnt pathway or for activation of cytokine-induced NF-κB pathway as well as the activation of AKT and ERK, the prediction calculation concludes in the function symbol in the nucleus. The predicted biological function in the selected pathways is indicated with the gear wheel-like symbols, pointing to migration, metastasis, anti-apoptosis, cell survival or cell proliferation.

The RNA-Seq datasets were transferred to the pathway analysis software Ingenuity Pathway Analysis (IPA), and the canonical pathway analysis tool revealed numerous canonical pathways (Supplemental Figure S1), with significantly activated patterns such as “acute phase response signaling” or presented in “colorectal cancer metastasis signaling” (Figure 9). Within this activated canonical pathway, the connections between activated or inhibited target molecules (receptors, protein kinases, phosphatases, transcription factors, etc.) are indicated by the blue or orange lines/arrows for the direction of inhibition or activation prediction between the target molecules. These pathway predictions confirmed our experimental findings and furthermore indicate connections between the experimentally observed polarity changes or cell detachment and the involved molecular targets. A colon carcinoma cell line, such as Caco-2, should already show baseline colorectal cancer signaling, but the comparison with the control condition in IPA will reveal changes in signaling, induced by infection with *E. coli* 536. In cancer signaling, the target molecules of PI3K/AKT were shown to be activated, and indeed in the IPA prediction, the whole canonical pathway of “PI3K/AKT signaling” was shown to be activated.

**Figure 10:**
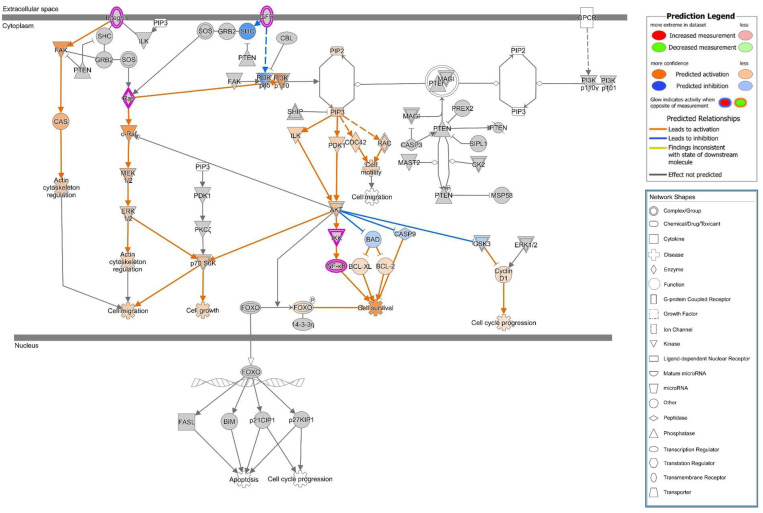
Canonical pathways inhibited in *E. coli* 536-infected Caco-2 monolayers. RNA-Seq and consecutive data analysis by IPA revealed “PTEN signaling” to be inhibited after infection. For identification of various symbols refer to the legend embedded into the figure. The schematic analysis reveals an overlay of our RNA expression dataset from what is overlayed onto the pre-existing signaling pathway. Regarding the arrows, orange color indicates activation, and blue color indicates inhibition. Grey nodes indicate no sufficient information. The pathway was visualized using the molecule prediction tool of IPA. Following the activation arrows, indicated for AKT or FAK with activated function (gear wheel-like symbols) of actin cytoskeleton regulation, cell migration, cell growth, cell survival or cell cycle progression. PIP3 points to CDC42 und Rac activation, promoting cell motility.

Even more interestingly, the canonical pathway “PTEN signaling” was predicted to be inhibited (Supplemental Figure S1). In the PTEN signaling pathway prediction, AKT shows an activation patterns, and PTEN itself is inconsistently regulated (Figure 10). These bioinformatic findings correspond to the observations made in our immunostainings with misregulated or inhibited PTEN activity (as shown above). Further information from activated molecules, e.g., “FAK affection” in this canonical pathway of PTEN inhibition point to cellular factors that will be substrate of future signaling studies uncovering the underlying signaling of the HlyA^+^ *E. coli* infection even further.

**Figure 11:**
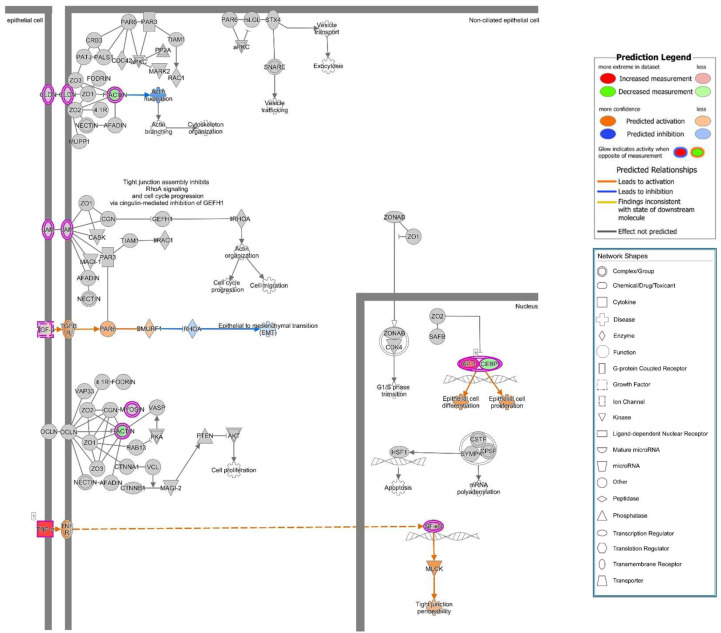
Canonical pathway affected in *E. coli* 536-infected Caco-2 monolayers. RNA-Seq and IPA of infected Caco-2 cells revealed “Tight junction signaling” to be affected after infection. Symbol legend incorporated into the figure. The dataset is overlayed onto a pre-existing signaling pathway. Orange color indicates activation, and blue color indicates inhibition. Grey nods indicate no sufficient information for a clear prediction. Affected or regulated nodes were visualized with the molecule prediction tool. TJ signaling showed no clear predictions in most nodes, but a hint to NF-κB-dependent MLCK (myosin light-chain kinase) signaling pointing to increased epithelial permeability was predicted in the TNF pathway.

Other barrier-relevant pathways are presented in Figure 11, revealing inconsistent effects on canonical pathways like “Tight Junction signaling” (Supplemental Figure S1), since the information from our datasets were not sufficient to make a broad prediction (grey nodes), but a useful result in this TJ pathway could nevertheless be seen by the activation of MLCK (myosin light-chain kinase, in the lower part of the image, Figure 11) with TNF-dependent activation of NF-κB, leading to increased epithelial permeability by actomyosin constriction and the retraction of TJ proteins from the membrane (redistribution and detachment of TJs). This finding goes in line with the redistribution of occludin identified in our immunostainings (Figure 4). However, this prediction method can only be used as hypothesis driver as further findings needed to be confirmed and proven by further cell biological experiments.

## 3. Discussion

In inflammatory or inflammation-prone conditions, HlyA was described as potentiator of the *leaky gut* phenomenon [4,7]. HlyA^+^ UPEC strains can also be found in the large intestine of animals or humans, even in healthy conditions [4]. In the phase of active inflammation in ulcerative colitis, they can be found in high numbers and in mouse models an infection with HlyA-producing *E. coli* 536 causes intestinal inflammation [4]. Whether or not they can play a role in human intestinal inflammation even without genetic predisposition remains unclear due to lack of study data. This can be explained partly because UPEC are not routinely assessed as, e.g., in infectious diarrhea, and partly because the nomenclature provokes confusion on different HlyA types, as the HlyA from UPEC is genetically different from the HlyA of enterohemorrhagic *E. coli* (EHEC). Although strong effects on epithelial cell shedding or barrier dysfunction of UPEC HlyA on polarized intestinal cells were known for many years, the mode of virulence of UPEC in the intestine remained unclear, especially on the level of cell signaling. In this study, we could provide new findings on the pathomechanisms of UPEC HlyA on the intestinal epithelium.

For bacterial adherence and invasion, a defined virulence factor of the UPEC pathovar are type-1 pili. The type-1 pilus adhesin FimH mediates bacterial invasion of human bladder epithelium [20]. In our live cell imaging analysis, we could show bacterial invasion into intestinal epithelial cells with both pili expressing *E. coli* 536 and the hemolysin-deficient mutant. Although invasion seems to be possible for UPEC strains even without HlyA, we could detect neither epithelial damage nor perturbation of the intestinal barrier after infection of Caco-2 cell monolayers. However, outer membrane proteins could have an effect on barrier function at later time points. Furthermore, incubation of sterile but HlyA-containing supernatant showed irreversible barrier-breaking effects on our cell monolayers, similar to infection with *E. coli* 536 [3,7]. Thus, the early effects of UPEC on intestinal cells are likely due to its secreted toxin HlyA. Moreover, this may depend on the bacterial secretion of HlyA and the localization of the bacteria in proximity to the epithelial cells. The inhibition experiment with mbCD showed that the insertion of the heptameric HlyA pore proteins into the host cell membrane is responsible for the barrier defect. Similarly, it was shown for HlyA and further bacterial PFTs like aerolysin as well as other PFTs that the cellular disturbance depends on a proper insertion of the bacterial pores into the host cell membranes. When pore formation is disturbed, e.g., by zinc ions or other cations, the subsequent cellular pathologies are blocked [6,7,21].

In previous studies on PIP distribution during bacterial infection on epithelial cells, both enteropathogenic *E. coli* (EPEC) and *Pseudomonas aeruginosa* caused transient focal PIP accumulations at the site of bacterial attachment [22,23]. In contrast to these observations, we did not see PIP accumulation in our infection model, not even after internalization of HlyA-negative HDM into Caco-2 cells. In our investigations, the PIP2 GFP-signal was eliminated from the membrane shortly after infection with *E. coli* 536. Thus, a different mechanism linked to PIP2 elimination seems to be involved into UPEC pathogenesis. The PTEN is a dual-specificity phosphatase. It was first recognized as a tumor suppressor in the 1990s [24], but nowadays, more and more of its wide range of biological functions are understood. The primary substrate of PTEN is PIP3, which is being hydrolyzed to PIP2 [25]. PIP3 recruits AKT to the membrane and leads to its activation. Thus, PTEN acts as an AKT antagonist. In our study, we found PTEN signals to be reduced in the apical cell membrane micro-domain after HlyA-infection both in Caco-2 and in GFP-PH-PLC-transfected Caco-2 cells. Moreover, an increased PTEN signal was observed inside the nuclei. This PTEN regulation is in line with the signaling predictions from IPA with AKT activation and PTEN inhibition. Furthermore, this dysregulation can be associated to the predicted activation of the metastasis signaling pathway.

PTEN also plays an important role in cell metabolism. Other PTEN substrates belong to the Src family and take part in the focal adhesion complex. In this context, PTEN is known to be an inhibitor of cell migration [26]. In fact, after PTEN inhibition with pbV(pic), we could observe increased numbers of cells that were detached from the monolayers. Infection with HlyA-secreting bacteria also leads to extensive detachment of cells from the tissue, morphologically similar to the bpV(pic)-induced cell exfoliation. In uroepithelial cells, this effect, called epithelial shedding, was previously described [16]. Furthermore, Dhakal and colleagues found the PTEN substrate FAK from the focal adhesion complex to be reduced after HlyA-infection [10], but to our knowledge, the present study is the first that links the HlyA effect to PTEN.

PTEN as a tumor suppressor belongs to the most frequently mutated genes in human cancers [13,24,26]. Loss of PTEN causes disruption in chromosomal structure and numerical chromosome instability and leads to cell cycle progression [24]. As reported, PTEN deficient intestinal cells have increased capacity to induce tumors [12]. Moreover, in our experiments on Caco-2 cells, PTEN localization was changed into the nuclei, and cell proliferation was increased after HlyA^+^ *E. coli* infection, pointing to changes in cell cycle regulation. Furthermore, after both HlyA^+^ *E. coli* infection and PTEN inhibition with bpV(pic), the cell shedding from the epithelium was promoted. In a parallel experiment, PTEN inhibition by bpV(pic) decreased epithelial resistance to the same extend as HlyA did. Thus, PTEN inhibition could mimic the barrier defect of HlyA. Nevertheless, whether the increase in proliferation was the result of a PTEN-driven effect or due to an activated restoration mechanism of the epithelium, is up to future investigations.

A previous study showed that upon suppression of PTEN, an up-regulation of ARF4 leads to altered expression of TJ proteins occludin and claudin-4 and increased epithelial permeability [27]. Unfortunately, we found no association of ARF4 to the infection in our RNA-Seq data. However, in our study, we observed a change of distribution of the AJ protein E-cadherin from cell–cell borders towards the cytosol after infection of Caco-2 cells with HlyA-positive *E. coli* 536. Under control conditions, PTEN and E-cadherin co-localize at the cell–cell border, and this interaction is a critical regulator of epithelial morphogenesis [28]. We could detect both PTEN and E-cadherin reduction from the apical cell-cell contacts after HlyA^+^ *E. coli* infection and measured a pronounced decrease in TER both after HlyA^+^ *E. coli* infection and after PTEN inhibition with bpV(pic). Since it was shown that changes in PTEN abundance in proximity to the apical membrane affect both AJ and TJ signaling, our results could be explained by toxin-induced disorganization of the barrier-forming junctional complexes. For the latter, we found evidence during the first events of TJ protein redistribution of occludin and ZO-1 in the bicellular TJ during cell detachment induced by HlyA within 3 h. Similar to this, we previously observed a subcellular redistribution of the TJ proteins occludin, claudin-4 and claudin-5 as well as a disorganization of E-cadherin and F-actin in colonic mucosae from pigs infected with *E. coli* 536 at 5 h after infection [6]. The first evidence for the initial detachment of the TJ protein occludin represents the start of the barrier dysfunction and also indicates the beginning of the cell detachment, which might be the initiation point of *focal leaks* and could end up in larger epithelial lesions, characteristic for the HlyA effect to induce *focal leaks* [3,4]. This HlyA-induced barrier dysfunction by *focal leak* formation was shown to be independent of apoptosis induction or TJ protein expression changes [3,4]. A profound change of TJ protein expression was not expected in the short incubation time of *E. coli* (4–5 h). In a previous study with orally *E. coli* 536-infected germ-free mice, claudin-1, -2 and -3 expression in the colonic mucosa was not changed even 3 days after infection. Hence, TJ expression regulation seems to play a minor role [4]. Thus, the TJ changes observed in the present study display the early events in cell loss towards *focal leak* formation as well as single cell shedding. These pathomechanisms point to the *leak flux* mechanism, the passive loss of water and solutes to the intestinal lumen, as type of diarrhea by HlyA-secreting *E. coli*. Further progression of the TJ disruption or *focal leak* induction contribute to the *leaky gut* phenomenon [4]. For future investigations, it will be worth to enlighten affected pathways contributing to the TJ and AJ detachment during the HlyA^+^ *E. coli* infection. In this context, studying MLCK signaling in dependence of NF-κB and TNF signaling or the calcium pathway after influx of Ca^2+^ ions through the HlyA pores would be as important as the investigation of the PI3K, AKT and PTEN pathway. However, Ca^2+^ pathways also activate other pathways, including Ca^2+^-dependent PKC isoforms, which were shown to rather strengthen barrier function in Caco-2 cells via the leukotriene pathway, and furthermore, the activation of prostaglandin signaling could lead to barrier dysfunction, both displaying further candidates in *E. coli* 536 pathology [29,30]. Nevertheless, the mechanisms behind cell extrusion by HlyA were characterized here for the first time. It starts with the detachment of TJs and progresses to cell separation and *focal leak* formation. First hints for signaling in this *focal leak* pathomechanism via cell detachment were shown here. However, further in vitro and in vivo experimentation and confirmation of the HlyA-induced pathways are needed to decipher the complete pathological process and its presumed role in colorectal cancer metastasis signaling.

A recent clinical observation study found *E. coli* HlyA and colibactin to be present in high abundance on colorectal cancer [31]. This observation, together with our experimental association of HlyA to the *leaky gut* with influx of noxious agents and to the pro-oncogenic PTEN inhibition as well as to the cell separation or even dissemination of colon carcinoma Caco-2 cells, could open new perspectives on the surveillance of these virulence factors in colorectal cancer and metastases. In future research approaches, a better understanding of the microbial influence on the progression of this disease could also lead to new treatment options. A recent study on serum levels of PTEN mRNA even suggested the use of PTEN as an efficient and independent diagnostic marker for colorectal cancer patients [32].

For future directions in medical research, it could be implemented that the colonization of the intestine with certain *E. coli* harboring hemolysin types like UPEC HlyA or other virulence factors such as colibactin could apply as prognostic factor in IBD or colorectal carcinoma, when these *E. coli* types are present in or around inflammatory foci or lesions and/or overgrowing neoplastic tissue.

This could have implications for a treatment option, e.g., with probiotics like *E. coli* Nissle or antibiotics against these bacteria or combination of such strategies, in order to attenuate the course of the disease.

## 4. Conclusions

From our data, we can conclude that HlyA induces PTEN inhibition leading to polarity changes via changing PIP2 to PIP3 ratio in the membrane. Concomitantly, early barrier dysfunction was measured that can be assigned to TJ and AJ redistribution and bicellular detachment between epithelial cells. Furthermore, cell exfoliation occurs, which likely could be assigned to the preceding cell detachment. In further progression, *focal leak* formation can be supported by the early mechanisms leading to cell loss. Moreover, cell detachment from the epithelium revealed a higher number of viable cells, which leave the cell association after HlyA treatment. The identified pathways associated with the HlyA effect combine different target molecules including Ca^2+^- or TNF-dependent MLCK activation, AKT activation and PTEN inhibition as well as cancer signaling. Thus, the pore-forming toxin α-hemolysin from *E. coli* did not simply induce cell death in intestinal epithelial cell but rather has a complex effect on host cell regulation, contributing to various pathologies including *leak flux* barrier dysfunction with *focal leak* formation potentiating antigen influx and the *leaky gut* phenomenon, propagating mucosal inflammation as well as tumorigenesis and metastasis.

In conclusion, the epithelial pathologies observed in this study are (i) cell polarity changes with PIP2 decrease; (ii) barrier dysfunction associated to PTEN dependent inhibition via TJ redistribution and (iii) viable cell shedding initially shown as cell junction detachment. All together promoting luminal antigen influx and pro-inflammatory or carcinogenic impact.

## 5. Materials and Methods

### 5.1. Bacterial Cultivation and Infection

Hemolysin-producing *E. coli* 536 and the *E. coli* 536 hemolysin-deficient mutant (HDM) (536ΔhlyI_ΔhlyII::cat) [33] were cultured at 37 °C overnight in Luria–Bertani broth. One hour before infection, the media on Caco-2 cells was replaced by a media not containing antibodies. An aliquot was brought to log phase (2 colonies in 10 mL culture medium at 37 °C and 220 rpm for 3 h) and a standard infection dose of 50 µL containing 10^5^ colony-forming units (CFU) was added to the apical compartment of epithelial cells, containing 500 µL of media, with a multiplicity of infection (MOI) of 100 to the epithelial cells. Cells were incubated with bacteria from 1 h up to 5 h.

### 5.2. Cell Culture

Human Caco-2 colon carcinoma cells [34] were cultured in liquid medium MEM- with Gutamax^TM^-1 (Gibco, Thermo Fisher Scientific Inc., Waltham, MA, USA) supplemented with 1% penicillin-streptomycin (P/S, Corning, Glendale, AZ, USA) and 15% fetal calf serum (FCS, Gibco, Thermo Fisher Scientific Inc., Waltham, MA, USA) in 25 cm^2^ culture flasks (Corning, Glendale, AZ, USA). The cells were maintained at 37 °C with 5% CO_2_ and were fed every two-days. After 14 days, Caco-2 cells reached confluency; they were washed with phosphate-buffered saline (PBS, Gibco, Thermo Fisher Scientific Inc., Waltham, MA, USA), incubated for 30 to 60 min at 37 °C with trypsin-EDTA solution (Sigma-Aldrich Chemie GmbH, Taufkirchen, Germany) and seeded on Millicell PCF filters (0.4 µm pore size, 12 mm Diameter, Merck Millipore ltd., Darmstadt, Germany). Cells were grown on filter supports for 10 to 14 days until forming a confluent monolayer before bacterial infection.

### 5.3. Transepithelial Resistance

The measurement of transepithelial electrical resistance (TER) of the colonic epithelial monolayers was performed as previously described [35]. A fixed pair of electrodes connected to an Ohmmeter (EVOM, WPI, Sarasota, FL, USA) was used to measure the transepithelial electrical resistance of confluent monolayers on filters, at a stable temperature of 37 °C. Correction values for the resistance of the empty filter and the area were considered. Resistance measurements were performed before as well as after infection with *E. coli* 536 and *E. coli* HDM or after inhibition of PTEN with bpV(pic) (1µM, Sigma-Aldrich Chemie GmbH, Taufkirchen, Germany), PI3K with Ly294002 (50µM, Cell Signaling Technology, Danvers, MA, USA), PLC with U73122 (5µM, Millipore, Merck KGaA, Darmstadt, Germany), MLCK with ML-7 or H-7 (Sigma-Aldrich Chemie GmbH, Taufkirchen, Germany), PKC with H7 or Calphostin C (Sigma-Aldrich Chemie GmbH, Taufkirchen, Germany) or ERK 1/2 with U0126 (Cell Signaling Technology, Danvers, MA, USA) from 1 h up to 5 h after infection or inhibition.

### 5.4. Transfection

Caco-2 cells were transiently transfected with the DNA construct PH-PLCδ-eGFP used as a reporter for PIP2. The constructs were obtained from Prof. Michael Krauß (FMP, Leibniz Forschungsinstitut für molekulare Pharmakologie, Berlin, Germany). Caco-2 cells were grown for 14 days and split and grown again for three days. After three days, the cells were trypsinized with 1 mL trypsin-EDTA for 10 min at 37 °C. The cells were counted, and the cells suspension concentration was adapted to reach 6 × 10^4^ cells/mL. A total of 750 000 cells were seeded in Lab-Tek II chambered coverglass well (0.7 cm^2^, Thermo Fisher Scientific Inc., Waltham, MA, USA), and cells were grown for 5 h in RPMI media + 10% FCS at 37 °C and 5% CO_2_. After 5 h, the DNA construct was added on the cells with media without FCS and Lipofectamin-2000 (Thermo Fisher Scientific Inc., Waltham, MA, USA). After 18 h of growth, media without P/S was added to fill the wells completely and after 24 h the media was changed for RPMI + 10% FCS. Two days after lipofection, cells were checked for successful transfection with GFP localized at the apical membrane of cells via LSM. Seven days after transfection, cells were infected with bacteria, and TER was measured before and throughout the infection period. For stable transfection, the same protocol was applied; however, two days after transfection, cells were checked for successful transfection, and G418 (Geneticin, PAN biotech, Aidenbach, Germany) was added on the cells at a concentration of 1000 µg/mL to select viable clones. After the non-transfected cells died, the concentration of G418 was reduced to 600 µg/mL, and successful clones were selected and grown separately in 24 wells plates until confluency. They were then transferred to 12-well plate, 6-well plate and finally 25 cm^2^ flasks. The successfully transfected clone #29 was selected and used for further experiments, and this Caco-2 cell line with stable GFP-PLC-PH expression formed a confluent epithelial cells monolayer after appropriate culturing. These polarized monolayers were able to establish an apical-basolateral barrier as measured with high transepithelial electrical resistances (TER).

### 5.5. Live Cell Imaging

PH-PLCdelta-eGFP-transfected Caco-2 cells were cultured in Lab-Tek wells or in inverted filter supports (0.4 µm pore size, 12 mm Diameter, Merck Millipore ltd., Darmstadt, Germany). Seven days after confluency, images were taken using a water immersion objective (LSM780, Zeiss, Jena, Germany). Incubation chamber under the LSM objective was heated to 37 °C, and cells were kept under constant 5% CO_2_ atmosphere in ambient air throughout the live cell imaging experiment. *E. coli* 536 and HDM were stained with Hoechst 33342 solution (dilution 0.67 µg/mL, Cell Signaling Technology, Danvers, MA, USA) for visualization and added to the cell filters with standard infection dose.

### 5.6. Visualization of E. coli HlyA and Epithelial Integrity by Immunofluorescence Staining

Caco-2 cell monolayers were fixed with 2% paraformaldehyde (Electron Microscopy Sciences, Hatfield, PA, USA). Then, cells were treated with 25 mM glycine (Carl Roth, Karlsruhe, Germany) in order to quench the protein linking up. TritonX-100 (Serva electrophoresis GmbH, Heidelberg, Germany) at 1% in phosphate-buffered saline with calcium and magnesium (PBS+, Gibco, Thermo Fisher Scientific Inc., Waltham, MA, USA) was used for permeabilization for 2 h and subsequently stopped with blocking solution (10% goat serum, 1% bovine serum albumin, 0.8% TritonX-100 in PBS+) for 3 h. The primary antibody was added in blocking solution overnight. Staining with a secondary IgG antibody (Alexa Fluor, Thermo Fisher Scientific Inc., Waltham, MA, USA) was performed after several washing incubations with PBS+ (Gibco, Thermo Fisher Scientific Inc., Waltham, MA, USA). DAPI (4’-6-diamidino-2-phenylindole dihydrochloride, Roche Diagnostics, Mannheim, Germany) and DY-647P1-Phalloidin (1:200 dilution, Dyomics, Jena, Germany) were used for staining nuclei and actin cytoskeleton, respectively, at room temperature for 30 min. The following antibodies were used: Occludin (rabbit, 1:200 dilution, Invitrogen, Thermo Fisher Scientific Inc., Waltham, MA, USA, no. 711500), ZO-1 (mouse, 1:100 dilution, BD Biosciences, San Jose, CA, USA, no. 610966), E-Cadherin (mouse, 1:200 dilution, BD Biosciences, San Jose, CA, USA, no. 610182), PTEN (rabbit, 1:100 dilution, Cell Signaling Technology, Danvers, MA, USA, no. 9559S), anti-human Ki-67 Alexa Fluor 647 (mouse, 1:200 dilution, Biolegend, San Diego, CA, USA, no. 350509).

### 5.7. Epithelial Cell Counting after Bacterial Treatment for Cell Separation Assay

Caco-2 monolayers were infected with *E. coli* 536 or control infection with HDM for 5 h; then, bacterial cells were inactivated by addition of 100 mg/L gentamicin (Carl Roth GmbH, Karlsruhe, Germany), and the cell culture medium (DMEM) was transferred as supernatant on fresh 24-well coated with 0.002% poly-L-lysine (Sigma-Aldrich Chemie GmbH, Taufkirchen, Germany), and the seeded Caco-2 cells were incubated for another 24 h in a cell culture incubator. The number of viable cells shedded from the infected Caco-2 monolayers was estimated the next day by microscopic counting of attached cells in a low-power field. Dead cells were counted microscopically by trypan blue staining and excluded from analysis.

### 5.8. Statistical Analysis

Data are expressed as mean values ± standard error of the mean (SEM). Statistical analysis was done with GraphPad Prism (GraphPad Software version 5, San Diego, CA, USA). Student’s *t*-test or 1-way ANOVA and *t*-test with Bonferroni correction was used for multiple comparisons. *p* < 0.05 was considered statistically significant.

### 5.9. Bioinformatic Pathway Analysis from RNA-Sequencing Expression Data

RNA was extracted from Caco-2 monolayers after 5 h incubation in an infection experiment with *E. coli* 536 (3 cell monolayer filters each) using the mirVana RNA Isolation Kit (Ambion, Life Technologies, Carlsbad, CA, USA). The following methods were described previously [36]. Briefly, library preparation of RNAs cDNA library and sequencing was performed by Illumina’s RNA-Seq prep kit following the manufacturer’s instructions. The purified DNA was quantified and diluted to 10 nM for cluster generation and sequencing on an Illumina NovaSeq6000 Sequencing System. The reads were mapped against the human genome GRCh37/hg19 using STAR Aligner v2.7.1a with double pass alignment [37]. First mapping was obtained using Ensembl v73 coordinates as a framework. Second-pass mapping added splice sites that were found in the first run. RNA-Seq expression analysis Gene-read coverages were obtained using Bioconductor package Rsubread [38]. The Bioconductor package DESeq2 was used to quantify the differential gene expression between two conditions in form of log2-fold changes with their corresponding *p*-values [39,40]. *p*-values were corrected for multiple testing according to Benjamini Hochberg to match the false discovery rate, and a cut-off of *p* < 0.05 (5% FDR) was chosen. Fastq files containing the raw reads from sequencing are available at Gene Expression Omnibus (GEO) archive under National Centre for Biotechnology Information (NCBI) website with GEO accession ID GSE169213.

Canonical pathway analysis was performed with the Ingenuity Upstream Regulator Analysis software (IPA, Qiagen Silicon Valley, Redwood City, CA, USA).

## Data Availability

RNA-Seq raw data are deposited in NCBI’s Gene Expression Omnibus under the GEO accession ID GSE169213. Further raw data supporting the conclusions of this article will be made available by the authors upon request.

## Supplementary Materials

The following are available online at https://www.mdpi.com/article/10.3390/toxins13080520/s1, Supplemental Figure S1: Canonical pathways in HlyA^+^ *E. coli* infection. List of the Canonical pathways in *E. coli* 536 infected Caco-2 cell monolayers 5 h p.i. From this complete list of affected pathways, we selected three canonical pathways to be presented in detail in the results Section 2.7.
